# Dengue Emergence and Adaptation to Peridomestic Mosquitoes

**DOI:** 10.3201/eid1010.030846

**Published:** 2004-10

**Authors:** Abelardo C. Moncayo, Zoraida Fernandez, Diana Ortiz, Mawlouth Diallo, Amadou Sall, Sammie Hartman, C. Todd Davis, Lark Coffey, Christian C. Mathiot, Robert B. Tesh, Scott C. Weaver

**Affiliations:** * University of Texas Medical Branch, Galveston, Texas, USA;; † Institut Pasteur, Dakar, Senegal

**Keywords:** dengue, arboviruses, Flaviviridae, mosquito, insect vectors, evolution, research

## Abstract

Phylogenetic evidence suggests that endemic and epidemic dengue viruses (DENV), transmitted among humans by the anthropophilic mosquitoes *Aedes aegypti* and *Ae. albopictus*, emerged when ancestral, sylvatic DENV transmitted among nonhuman primates by sylvatic *Aedes* mosquitoes adapted to these peridomestic vectors. We tested this hypothesis by retrospectively examining evidence for adaptation of epidemic and endemic versus sylvatic strains of DENV-2 to *Ae. albopictus* and *Ae. aegypti*. First and second-generation offspring of mosquitoes from different geographic regions in the Americas and Southeast Asia were tested for their susceptibility to epidemic/endemic and sylvatic DENV-2 isolates from West Africa, Southeast Asia, and Oceania. Both *Aedes* species were highly susceptible (up to 100% infected) to endemic/epidemic DENV-2 strains after ingesting artificial blood meals but significantly less susceptible (as low as 0%) to sylvatic DENV-2 strains. Our findings support the hypothesis that adaptation to peridomestic mosquito vectors mediated dengue emergence from sylvatic progenitor viruses.

## Abstract 2

Endemic dengue virus (DENV) type 2 strains infect *Aedes aegypti* and *Ae. albopictus* more efficiently than ancestral sylvatic strains, which suggests that adaptation to these vectors mediated DENV emergence.

Dengue is caused by any of four antigenically distinct serotypes of dengue virus (DENV), family *Flaviviridae*. An estimated 100 million annual dengue cases occur each year in tropical cities, in which more than 2.5 billion people (almost half of the global population) are at risk (*1*). Infection with one DENV serotype confers lifelong protection against homologous reinfection, while a subsequent heterologous infection increases the likelihood of a more severe form of the disease (*2**–**4*).

Dengue has four clinical manifestations: 1) undifferentiated illness, 2) classic dengue fever, 3) dengue hemorrhagic fever, and 4) dengue shock syndrome. Undifferentiated dengue, the most common syndrome, occurs when a DENV infection is asymptomatic or mildly symptomatic. Dengue fever involves an abrupt febrile illness lasting 2–7 days, accompanied by malaise, headache, retroorbital pain, myalgia, and arthralgia of such great intensity that it has earned the lexicon "break-bone" fever (*5**,**6*). Dengue hemorrhagic fever progresses to hemorrhagic manifestations and plasma leakage caused by increased vascular permeability. Dengue shock syndrome is characterized by circulatory failure and is the most lethal dengue syndrome (*7*).

Within forest habitats of West Africa, Malaysia, and probably Vietnam, zoonotic, sylvatic dengue cycles have been described involving *Aedes* spp. mosquitoes and monkeys (*8**–**10*). Sylvatic DENV vectors in Africa include *Aedes* (*Stegomyia*) *africanus*, *Ae.* (*S.*) *luteocephalus*, *Ae.* (*S.*) *opok*, *Ae. (Diceromyia) taylori*, and *Ae.* (*D.*) *furcifer* (*10*); in Malaysia, *Ae. niveus* has been implicated in transmission (*8**,**9*). These sylvatic cycles, probably involving only DENV-2 in West Africa but all four serotypes in Malaysia, are believed to represent the ancestral DENV cycles from which epidemic/endemic (henceforth referred to as endemic) strains of DENV-1–4 evolved independently hundreds to thousands of years ago (*11*). Although humans occasionally become infected with sylvatic DENV in West Africa and perhaps in Asia, they are tangential to the maintenance cycle, which involves sylvatic *Aedes* spp. mosquito vectors and nonhuman primates as reservoir hosts. In contrast to the sylvatic cycles, epidemic DENV cycles involving transmission among humans by *Ae.* (*Stegomyia*) *aegypti*, *Ae.* (*S*.) *albopictus*, and other anthropophilic *Aedes* species have emerged in large tropical urban cities (*12*). These urban cycles are ecologically and evolutionarily independent of the ancestral sylvatic cycles, with humans serving as reservoir hosts.

Dengue is a reemerging disease in the neotropics and is transmitted primarily by *Ae. aegypti*. The abundance in Africa of closely related *Aedes* species within the *Stegomyia* subgenus, the lack of closely related *Stegomyia* species in the Americas, and the existence of sylvatic *Ae. aegypti* (*Ae. aegypti formosus*) in Africa suggest an African origin for this species (*13**–**17*). Movement of people and their requisite water storage containers during the 17th to 19th centuries probably spread *Ae. aegypti* throughout the tropics and subtropics. After World War II, *Ae. aegypti* prevalence and distribution increased in Asia and the Pacific Islands. *Ae. aegypti* was partially eradicated from tropical America in the 1940s and 1950s, but peridomestic *Ae. aegypti aegypti* has now reinfested most of the neotropics (*12*).

The Asian tiger mosquito, *Ae. albopictus*, originally of sylvatic origin as well, has spread widely in the world since the 1970s, including to the United States, Latin America, tropical Africa, the Pacific Islands, and Europe (*7*). Although less anthropophagic than *Ae. aegypti,* it is a secondary vector of DENV and possibly of greater importance in the early historical stages of urban dengue emergence.

We hypothesized that all four endemic dengue viruses evolved independently from sylvatic progenitors by adaptating to peridomestic mosquito vectors and human reservoir hosts (*11*). The rise of urban civilizations and the associated peridomestication of *Ae. aegypti* and *Ae. albopictus* mosquitoes provided this opportunity for adaptation and resulted in the emergence of dengue in urban areas of the tropics. This hypothesis predicts that endemic DENV strains are more efficient at infecting urban mosquitoes such as *Ae. aegypti* and *Ae. albopictus* than are the ancestral, sylvatic DENV strains. We tested this hypothesis by using experimental infections of *Ae. aegypti* and *Ae. albopictus* with sylvatic versus urban strains of DENV-2. Our results support the hypothesis that adaptation to peridomestic mosquito vectors mediated dengue emergence from sylvatic progenitor viruses.

## Methods

### Mosquito Colonies

Because geographic variation exists with regard to susceptibility to DENV in both colonized (*18**,**19*) and wild-collected populations of *Ae. aegypti* and *Ae. albopictus* (*20**–**22*), mosquitoes from the United States, Brazil, Bolivia, and Thailand were tested. These locations were selected to represent a wide geographic range, including regions with endemic dengue, and on the basis of availability from specimens from collaborators. Because laboratory colonization has been shown to affect susceptibility of mosquitoes to oral infection by flaviviruses (*23**,**24*), low filial generation cohorts were used for susceptibility experiments. *Ae. aegypti* and *Ae. albopictus* females were collected during the fall of 2001 from Galveston, Texas, and the first filial (F1) laboratory generation was used for experiments. F1 generation *Ae. aegypti* females were also hatched from eggs collected in Mae Sed, Tak, Thailand, in 2002. Second generation (F2) *Ae. aegypti* collected in Santa Cruz, Bolivia, in 2002 were also used. From Brazil, F1 *Ae. albopictus* from Pindamonhangaba City (an urban environment) and F1 and F2 *Ae. albopictus* from Pedrinhas City (a rural environ) were used from a parental collection in 2001. All mosquitoes were maintained in an insectary at 28°C, with a relative humidity of 80% and a 12:12 light-dark circadian cycle. Adults were fed a hamster blood meal to obtain eggs. Eggs were stored in plastic containers for up to 3 months. Larvae were reared on a diet of ground rabbit and mouse chow. Pupae were transferred to screened cages, and adults were fed 10% sucrose ad libitum.

### Virus Strains

Low-passage isolates of DENV-2 were selected for this study to represent similar geographic ranges for endemic and sylvatic strains and based on the ability to obtain high-titered stocks after passage in mosquito cell (C6/36) cultures (Table 1); the strains included endemic strains New Guinea C (prototype strain) and 1349 and sylvatic strains PM 33974, A2022, and P81407. Virus stocks were prepared on C6/36 cell cultures and quantified by infecting C6/36 cells in 96-well plates with serial dilutions, followed by cell spotting in 12-well slides and immunofluorescence assays (IFA) to determine 50% tissue culture infective doses (TCID_50_) (see below). All work with DENV was carried out in a biosafety level 2 laboratory at the University of Texas Medical Branch with recommended safety procedures (*25*).

**Table 1:** DENV-2 strains used in this study^a^

Virus strain	Virus type	Host	Passage history^b^	Blood meal titer (log_10_TCID_50_/mL)	Location	Year
1349	Endemic	Human	Mosquito 2, C6/36 2	6.5	Burkina Faso (Upper Volta)	1982
New Guinea C	Endemic	Human	Monkey 1, mosquito 4, C6/36 1	8	New Guinea	1944
PM33974	Sylvatic	*Aedes africanus*	*Toxorhynchites amboinensis* 1, C6/36 2	8	Guinea	1981
DAK AR 2022	Sylvatic	*Ae. africanus*	SM6, C6/36-2	10	Burkina Faso (Upper Volta)	1980
P8-1407	Sylvatic	Sentinel monkey	SM3, C6/36-2	9.5	Malaysia	1970

^a^DENV, dengue virus; TCID_50_, 50% tissue culture infective dose; SM, suckling mouse. ^b^C6/36, *Ae. albopictus* cell culture.

### Indirect Fluorescent Antibody Test

Infection of mosquitoes with DENV was assayed with IFA in C6/36 cells, which is more sensitive than direct IFA of mosquito tissues (data not shown). Mosquito bodies and legs were triturated in Eagle's minimal essential medium (MEM) supplemented with 10% fetal bovine serum, 1% nonessential amino acids, glutamine, and antimicrobial agents (penicillin and streptomycin). Ten microliters of each triturated suspension was added to 90 µL of MEM in 96-well microtiter plates. Plates were incubated for 7 days at 28°C. After incubation, 10 µL suspensions of C6/36 cells were placed on multiwell slides, air dried, and fixed in ice-cold 80% acetone. Slides were then incubated for 1 h at 37°C with a polyclonal anti-DENV-2 mouse ascitic fluid diluted 1:80 in phosphate-buffered saline (PBS). Slides were rinsed twice in PBS and overlaid with fluorescein isothiocyanate–labeled goat anti-mouse immunoglobulin G (Sigma, St. Louis, MO) diluted 1:15 in PBS. Slides were again incubated for 1 h at 37°C and washed twice in PBS. Slides were examined at 200 to 1,000x with an inverted fluorescent microscope.

### Vector Susceptibility

Artificial blood meals consisting of 1% sucrose, 20% fetal bovine serum, 5 mmol ATP, 33% PBS-washed sheep blood cells, and 33% MEM were used for mosquito susceptibility determinations. Multiple cohorts of 30 to 50 mosquitoes were offered blood meals incubated at 37°C in a water-jacketed membrane feeder (*23*). After 1 h of feeding, engorged mosquitoes were sorted from unengorged ones, and a sample of the blood meal was assayed to determine the virus titer. Fully engorged mosquitoes were incubated for 14 days at 27°C with a 12:12 light-dark cycle. Then, legs were detached from cold-anesthetized mosquitoes and assayed to determine the dissemination rate of the virus from the midgut into the hemocoel (mosquito legs include hemolymph, which is believed to mediate infection of the salivary glands). Bodies were assayed to determine the overall infection rate. Blood meal titers were determined by IFAs on C6/36 cells (see above) of samples collected immediately after mosquito feeding.

Because mosquito infections caused by artificial blood meals are inefficient compared to those using viremic hosts, we used the highest virus titers available (6.5–10.0 log_10_ TCID_50_/mL) from cell culture passages for our experiments. To interpret our data as conservatively as possible, infection and dissemination rates for endemic strains were only compared with rates from the same or higher blood meal titers for sylvatic strains. Infection and dissemination differences were tested for significance with chi-square and Fisher exact tests with the SPSS (Chicago, IL) Base 11.5 statistical package. When no significant differences were observed among endemic or sylvatic strains tested, data were pooled for each group.

## Results

### *Aedes aegypti* Susceptibility

After ingestion of artificial blood meals containing 6.5–8.0 log_10_ TCID_50_/mL of endemic DENV-2, infection and dissemination rates in *Ae. aegypti* mosquitoes from Galveston, Texas, were 86.5%–100.0% and 90.6%–78.9%, respectively (Table 2). Infection and dissemination rates after exposure to sylvatic viruses were more variable but lower, ranging from 11.4% to 69.4% and from 0% to 64.4% after ingestion of 8.0 to 10.0 log_10_ TCID_50_/mL of DENV. Even after the (lowest) infection rate data for strain 1407 were removed from the pooled analysis because they were significantly different than those for the other sylvatic strains, both infection (p < 0.0001) and dissemination (p = 0.01) rates were different between endemic and sylvatic strains (Table 2).

**Table 2:** DENV-2 infection and dissemination rates in *Aedes aegypti*, Galveston^a,b^

Dengue strain	% infected (totals)	% dissemination^c^ (totals)
1349 (endemic)	86.5 (32/37)	90.6 (29/32)
New Guinea C (endemic)	100 (38/38)	78.9 (30/38)
33974 (sylvatic)	54.2 (26/48)	61.5 (16/26)
2022 (sylvatic)	69.4 (25/36)	64 (16/25)
1407 (sylvatic)	11.4 (4/35)	0 (0/4)
Collapsed^d^		
Endemic	93.3 (70/75)	84.3 (59/70)
Sylvatic	63.0 (51/81)	62.7 (32/51)

^a^DENV, dengue virus. ^b^Blood meal titers are found in Table 1. ^c^Number of infected mosquitoes with virus in the legs. ^d^Strain 1407 data were not included in the collapsed analysis because they were significantly different from data for other sylvatic strains.

*Ae. aegypti* from Santa Cruz, Bolivia, had lower infection and dissemination rates than Galveston populations after being exposed to endemic DENV strains. Infection rates were 40.9%–48.1% and dissemination rates were 76.9%–77.8% with blood meal titers of 9.5 log_10_ TCID_50_/mL (Table 3). Infection and dissemination rates in Bolivian mosquitoes exposed to sylvatic viruses were also lower than those of *Ae. aegypti* from Galveston, and sylvanic strain infection rates in Bolivian mosquitoes were lower (p = 0.015), ranging from 16.7% to 27.3%, than those of endemic strains; dissemination rates were not significantly different (p = 0.663).

**Table 3:** DENV-2 infection and dissemination rates in *Aedes aegypti*, Bolivia^a,b^

Dengue strain	% infected (totals)	% dissemination^c^ (totals)
1349 (endemic)	48.1 (13/27)	76.9 (10/13)
New Guinea C (endemic)	40.9 (27/66)	77.8 (21/27)
33974 (sylvatic)	16.7 (4/24)	100 (4/4)
2022 (sylvatic)	27.3 (6/22)	83.3 (5/6)
Collapsed		
Endemic	43 (40/93)	77.5 (31/40)
Sylvatic	21.7 (10/46)	90 (9/10)

^a^DENV, dengue virus. ^b^Blood meal titers are found in Table 1. ^c^Number of infected mosquitoes with virus in the legs.

*Ae. aegypti* from Mae Sed, Tak, Thailand, were also less susceptible than the Galveston population to DENV-2 strains used in this study, with the exception of endemic DENV-2 strain 1349 from Burkina Faso (infection and dissemination rates were 94.3% and 80%, respectively, with this strain) (Table 4). Like the Galveston and Bolivian populations, the Thai population exhibited consistent differences in susceptibility to endemic versus sylvatic strains (33.0%–94.3% infection with the endemic strains vs. 0%–13% for sylvatic strains; 84.8%–90.9% dissemination rate for endemic strains vs. 0%–50% for sylvatic strains). Infection rates for both endemic strains were higher than for the pooled sylvatic rates (p < 0.001), while dissemination rates were not significantly different (p > 0.1).

**Table 4:** DENV-2 infection and dissemination rates and *Aedes aegypti*, Thailand^a,b^

Dengue strain	% infected (totals)	% dissemination^c^ (totals)
1349 (endemic)	94.3 (33/35)	84.8 (28/33)
New Guinea C (endemic)	33 (11/33)	90.9 (10/11)
33974 (sylvatic)	13 (6/46)	50 (3/6)
2022 (sylvatic)	8.1 (3/37)	0 (0/3)
1407 (sylvatic)	0 (0/27)	0 (0/0)
Collapsed^d^		
Sylvatic	8.2 (9/110)	33.3 (3/9)

^a^DENV, dengue virus. ^b^Blood meal titers are found in Table 1. ^c^Number of infected mosquitoes with virus in the legs. ^d^Data for the two endemic strains were not collapsed because they were significantly different.

### *Ae. albopictus* Susceptibility

Like *Ae. aegypti*, *Ae. albopictus* from Galveston, Texas, exhibited greater susceptibility to endemic than sylvatic DENV strains. After ingesting blood meals containing 6.5–8.0 log_10_ TCID_50_/mL of endemic strains, 92.3%–100% of mosquitoes became infected, with high rates of dissemination (Table 5). In contrast, only 11.1% of mosquitoes became infected after ingesting 8.0 log_10_ TCID_50_/mL of the sylvatic strain. The infection rates for endemic strains were higher than for sylvatic strains (p < 0.0001), but the difference in dissemination was not significant (p = 1.000; only one infected mosquito with a sylvatic strain was tested and exhibited dissemination).

**Table 5:** DENV-2 infection and dissemination rates in *Aedes albopictus*, Galveston^a,b^

Dengue strain	% infected (totals)	% dissemination^c^ (totals)
1349 (endemic)	100 (12/12)	91.6 (11/12)
New Guinea C (endemic)	92.3 (12/13)	75 (9/12)
33974 (sylvatic)	11.1 (1/9)	100 (1/1)
Collapsed		
Endemic	96 (24/25)	83.3 (20/24)
Sylvatic	11.1 (1/9)	100 (1/1)

^a^DENV, dengue virus. ^b^Blood meal titers are found in Table 1. ^c^Number of infected mosquitoes with virus in the legs.

*Ae. albopictus* from two different locations in Brazil were also tested. One location was urban, Pindamonhangaba, while the other, Pedrinhas, was rural. Again, a significant difference in susceptibility was observed between endemic (strain New Guinea C) and sylvatic (strain 33974) infections with F1 mosquitoes of both geographic locations (Pindamonhangaba, p < 0.001, Pedrinhas, p < 0.001). However, no significant differences were detected in dissemination rates for either F1 mosquito population (p > 0.1). For the Pedrinhas mosquito population, F2 mosquitoes were tested with additional endemic and sylvatic strains. Again, the endemic strains infected at a higher rate (p < 0.001) than sylvatic strains, even when blood meal titers were lower (Table 6). Dissemination rates were also markedly different (p = 0.004).

**Table 6:** DENV-2 infection and dissemination rates in *Aedes albopictus* (Brazil)^a,b^

Geographic population, generation	Dengue strain	% infected (totals)	% dissemination^c^ (totals)
Pindamonhangaba F1			
	New Guinea C (endemic)	76.9 (10/13)	90 (9/10)
	33974 (sylvatic)	10.7 (3/28)	100 (3/3)
Pedrinhas F1			
	New Guinea C (endemic)	100 (10/10)	100 (10/10)
	33974 (sylvatic)	10 (2/20)	50 (1/2)
Pedrinhas F2			
	1349 (endemic)	100 (17/17)	88 (15/17)
	New Guinea C (endemic)	95.7 (22/23)	100 (22/22)
	33974 (sylvatic)	46.2 (6/13)	100 (6/6)
	2022 (sylvatic)	0 (0/15)	0 (0/0)
	1407 (sylvatic)	33.3 (5/15)	0 (0/5)
Collapsed (Pedrinhas F2)^d^			
	Endemic	49.3 (39/79)	94.9 (37/39)
	Sylvatic	21.4 (6/28)	100 (6/6)

^a^DENV, dengue virus; F2, second generation. ^b^Blood meal titers are found in Table 1. ^c^Number of infected mosquitoes with virus in the legs. ^d^Strain 2022 data were not included in the collapsed analysis because they were significantly different than data for the other sylvatic strains.

### Geographic Variation in Susceptibility among Mosquito Populations

Geographic variation for DENV susceptibility has been reported previously for both *Ae. aegypti* and *Ae. albopictus* (*18**,**19*). We found geographic variation among populations of both species. In general, *Ae. aegypti* from Galveston, Texas, were more susceptible than those from Bolivia (p < 0.001) but not those from Thailand (p > 0.1). *Ae. albopictus* from Galveston were also more susceptible to DENV-2 infection than those collected in Brazil (p = 0.009).

### Overall Trends

In general, in the 701 peridomestic mosquitoes from four localities used in this study, we found high susceptibility to endemic DENV-2 isolates but much less susceptibility to sylvatic strains. These differences were detected despite the blood meal titers of sylvatic strains being equal to or greater than those of endemic strains. Dissemination rates within infected mosquitoes generally showed no significant difference between endemic and sylvatic strains. Our data also indicated that *Ae. albopictus* was more susceptible to endemic DENV-2 strains than *Ae. aegypti* (overall 94% vs. 69% infection rates). Comparing *Ae. aegypti* and *Ae. albopictus* from one geographic location, Galveston, we did not find a difference between mosquito species when we compared infection with endemic strains 1349 (p > 0.1) or the New Guinea C strain (p > 0.1). However, Galveston *Ae. aegypti* were more susceptible to sylvatic strain 33974 than were *Ae. albopictus* from Galveston (p = 0.026).

## Discussion

### Historical Emergence of Dengue and Adaptation to Peridomestic Vectors

Our findings support the hypothesis that endemic DENV-2 strains are more efficient than sylvatic strains at infecting the peridomestic DENV vectors *Ae. aegypti* and *Ae. albopictus*. The overall trend that endemic DENV-2 strains were consistently more efficient at infecting peridomestic *Aedes* mosquitoes than were sylvatic DENV-2 strains (p = 0.000) supports our central hypothesis. Our data and previous phylogenetic studies (*11*) suggest that the emergence of endemic DENV from sylvatic progenitor strains occurred in conjunction with the peridomestication of *Aedes* mosquitoes and virus adaptation to these anthropophilic vectors. Although we tested only DENV-2 strains, emergence of DENV serotypes 1, 3, and 4 may also have been mediated by vector switching (from infecting sylvatic *Aedes* mosquitoes to *Ae. aegypti* and *Ae. albopictus*). Very few sylvatic DENV-1 and 4 strains are available (and none of DENV-3), which makes evaluating this hypothesis difficult.

The four independent evolutionary DENV emergence events (DENV-1–4) suggest that adaptation of DENV to new vectors and hosts occurred repeatedly from 300 to 1,500 years ago in Asia or Oceania (*11**,**26*). Since *Ae. aegypti* is not thought to have inhabited these regions at that time, *Ae. albopictus* was probably the original human vector (*12*). The widespread importance of *Ae. aegypti* as a vector may have begun in the 1700s, as commercial and slave trade transported it from its African origin. DENV-2 was probably introduced into Africa from Asia-Oceania approximately 1,000 years ago (*11*). The hypothesis that *Ae. albopictus* was the original peridomestic vector was supported by our study; *Ae. albopictus* was more susceptible to endemic DENV-2 strains than *Ae. aegypti*. The greater overall susceptibility (regardless of geographic origin) of *Ae. albopictus* compared to *Ae. aegypti* (94% and 69%, respectively) suggests a higher degree of adaptation, representing longer historical contact with *Ae. albopictus*. Other studies with sympatric populations from Brazil show *Ae. aegypti* to be more susceptible than *Ae. albopictus* to endemic DENV-2 (*19**,**20*).

### Risk for Dengue in the United States

When the vectorial capacity of a mosquito for an arbovirus is considered, many factors come into play, including mosquito survivorship, density, proportion of infected mosquitoes that are feeding, extrinsic incubation period, vector susceptibility, and density of susceptible hosts (*27*). We used vector susceptibility in this study as a measure not only of epidemiologic importance but also of the extent of adaptation of a virus to its vector. However, the full competence of a vector is established not only by its ability to become infected but also by its ability to transmit a pathogen. This feature is what gives vector competence its epidemiologic importance. In our study, transmission potential was estimated from dissemination rates because previous studies have suggested that mosquitoes are capable of transmitting DENV as long as the virus is able to disseminate from the midgut into the hemocoel (i.e., there is no evidence of a salivary gland infection barrier) (*18*). Mosquitoes that have a disseminated infection were therefore assumed to be capable of transmission.

Current methods of dengue control rely primarily on mosquito control and are aimed at reducing the populations of urban vectors, especially *Ae. aegypti*. This mosquito was eradicated from much of the New World during the middle of the 20th century. After the termination of the *Ae. aegypti* eradication program, *Ae. aegypti* populations reinfested many of the New World countries from which they had been eliminated, probably from those that did not achieve eradication. Being well adapted to urban environments and competent for transmission, DENV has become the most important mosquitoborne virus in the neotropics. Air travel and migration have increased the movement of virus strains around the world. Dengue virus has frequently been imported into the United States, where local transmission has been reported (*28*). Much of the southern United States is at risk for dengue transmission because of the presence of endemic *Ae. aegypti* and *Ae. albopictus*. Our study suggests that local populations of both species from Galveston are highly susceptible and potentially able to transmit DENV-2 from Africa, Asia, and Oceania.

### Implications for Dengue Control

Promising candidate dengue vaccines are raising hopes of effectively preventing human disease (*29*). Because humans are the only reservoir host for the endemic cycle, an effective vaccine could ultimately eradicate endemic strains. This scenario underscores the need for greater understanding of the historical emergence of human dengue from sylvatic origins to predict the facility with which the sylvatic strains could reemerge to initiate urban transmission. The four independent emergence events (DENV-1–4) suggest that the host-range changes that accompanied emergence can be readily accomplished by DENV; however, this hypothesis needs to be tested experimentally. One question to be answered is how many mutations are responsible for the efficient infection phenotype for *Ae. aegypti* and *Ae. albopictus* exhibited by the endemic DENV-2 strains. Identifying genetic determinants of DENV adaptation to these peridomestic vectors will ultimately provide an indication of the ability of these arboviruses to reemerge.

The viral molecular determinants that confer DENV with the ability to infect and be transmitted by their mosquito vectors are not known. Phylogenetic studies suggest that the DENV E protein may be important in the adaptation to urban vectors (*11*). In particular, domain III of the E protein contains several hypothetical amino acid replacements associated with emergence of urban strains. This clustering of changes in domain III is observed repeatedly during the emergence of DENV-1, DENV-2, and DENV-4, when phylogenetic methods are used. The envelope glycoproteins of other mosquitoborne viruses, including Sindbis (*30*), Venezuelan equine encephalitis (*31**–**33*), and La Crosse viruses (*34*), have been shown to mediate vector infection. Another genomic region potentially important in mediating vector transmission may be the 5´ noncoding region. Deletions in this region of DENV-4 constrain its ability to infect *Ae. aegypti* and *Ae. albopictus* mosquitoes (*35*).

Our study examined the extent of endemic DENV adaptation to peridomestic vectors. If this adaptation is species-specific, then sylvatic vectors may be more susceptible to infection by sylvatic than endemic DENV strains. We are currently evaluating this hypothesis with sylvatic West African vectors.
